# Logical modelling uncovers developmental constraints for primary sex determination of chicken gonads

**DOI:** 10.1098/rsif.2018.0165

**Published:** 2018-05-23

**Authors:** Lucas Sánchez, Claudine Chaouiya

**Affiliations:** 1 Dpto. Biología Celular y Molecular, Centro de Investigaciones Biológicas (C. S. I. C.), Ramiro de Maeztu 9, 28040 Madrid, Spain; 2 Instituto Gulbenkian de Ciência – IGC, Rua da Quinta Grande, 6, 2780-156 Oeiras, Portugal

**Keywords:** primary sex determination, chicken, gene regulatory network, logical modelling

## Abstract

In the chicken, sex determination relies on a ZZ (male)/ZW (female) chromosomal system, but underlying mechanisms are still not fully understood. The Z-dosage and the dominant W-chromosome hypotheses have been proposed to underlie primary sex determination. We present a modelling approach, which assembles the current knowledge and permits exploration of the regulation of this process in chickens. Relying on published experimental data, we assembled a gene network, which led to a logical model that integrates both the Z-dosage and dominant W hypotheses. This model showed that the sexual fate of chicken gonads results from the resolution of the mutual inhibition between DMRT1 and FOXL2, where the initial amount of DMRT1 product determines the development of the gonads. In this respect, at the initiation step, a W-factor would function as a secondary device, by reducing the amount of DMRT1 in ZW gonads when the sexual fate of the gonad is settled, that is when the SOX9 functional level is established. Developmental constraints that are instrumental in this resolution were identified. These constraints establish qualitative restrictions regarding the relative transcription rates of the genes DMRT1, FOXL2 and HEMGN. Our model further clarified the role of OESTROGEN in maintaining FOXL2 function during ovary development.

## 1. Introduction

In chickens, a reference experimental model, sex determination is genetically determined, that is individual sex results from the chromosomal constitution established in the zygote at fertilization. Chicken sex chromosomes, constituted by a ZZ (male) or a ZW (female) system, are unrelated to the XX/XY mammalian chromosomes [1]. The ZZ male embryo shows two functional symmetrical testes; whereas the ZW female embryo shows gonadal asymmetry, in which the left gonad leads to a functional ovary, whereas the right gonad starts growing to finally degenerate [2].

This work focuses on the sexual development of the chicken bi-potential gonad into either testis or ovary. In the past years, molecular genetic technologies have permitted the cloning and characterization of genes controlling primary sex determination. These genes can be categorized into two classes, male- and female-promoting genes, depending on their role in controlling the development of bi-potential gonads (see electronic supplementary material, figure S1).

Currently, the Z-dosage and dominant W-chromosome scenarios constitute the main propositions to explain chicken sex determination. The Z-dosage hypothesis argues that the sexual development of the gonads is dictated by a Z-linked gene, which is present at higher doses in ZZ gonads (male development) than in ZW gonads (female development). This would preclude a dosage compensation for this hypothetic Z-linked gene. In this respect, DMRT1 appears to be the best candidate as it fulfils a series of criteria required for a sex determination primary signal. First, DMRT1 is Z-linked being thus present in two doses in males and in one dose in females. Second, DMRT1 is specifically expressed in the gonads of chicken embryos, with a higher expression in males than in females [3,4]. Third, DMRT1 expression similar to that observed in ZZ males has been documented in female-to-male sexual reversed ZW chicken embryos [5]. Fourth, elimination of DMRT1 function in ZZ males causes male-to-female sex reversal [6].

On the other hand, the hypothesis of a dominant W-chromosome asserts the existence of a W-linked gene whose expression would determine female development, and whose absence in ZZ gonads would lead to testis development. Two W-linked genes have been identified as potentially involved in chicken sex determination. One is WPKCI (also known as ASW or HINTW), which exists in many copies on the W chromosome, whereas a single copy (ZPKCI) is present on the Z chromosome [7,8]. However, because ectopic expression of WPKCI in ZZ embryos does not cause gonadal sex reversal, this gene cannot be considered as a *bona fide* primary sex determination [9]. Nor does FET1 (female-expressed transcript 1), another W-linked gene whose expression is asymmetric, i.e. observed only in the left gonad [10]. Nevertheless, considering that intersexual ZZW chickens, as described by Thorne *et al.* [11] and Lin *et al.* [12], develop right testes and left ovotestes, a potential role of the W chromosome cannot be excluded.

In mammals, sex determination results from two consecutive processes: (i) *primary sex determination*, which refers to the development of bi-potential gonads towards either male (testis) or female (ovary) pathways; and (ii) *secondary sex determination*, which refers to the development of the sexual dimorphic structures, directed by sex hormones produced in the differentiated gonads [13,14]. Sex reversal can be experimentally induced in chickens by oestrogen administration or production disturbance, indicating a role of this hormone in avian sex determination [15]. Vertebrate bi-potential gonads—along the evolutionary transition from lower to higher vertebrates—seem to display a gradual resistance to sex hormones. Indeed, gonadal sex differentiation in fish [16] and amphibians [17] is generally susceptible to androgens and oestrogens; in reptiles [18], birds [15] and marsupials [19]; it is susceptible to oestrogens but not to androgens, whereas in eutherian mammals, gonadogenesis is resistant to hormone treatment [20].

Recently, logical modelling and formal analyses of the gene network underlying sex determination in placental mammals have been undertaken. The work of Ríos *et al.* [21] shows that, despite the likely involvement of a greater number of players, a core regulatory network seems enough to drive this complex developmental process. The authors further pointed to ß-CATENIN requirement for female development and its putative role in regulating FOXL2. Furthermore, our own modelling work considering a core regulatory network indicates that the gonad sexual fate results from the sequential resolution of two connected feedback loops: the mutual inhibition of SOX9 and ß-CATENIN at an initiation phase that subsequently affects the mutual inhibition between DMRT1 and FOXL2, at a maintenance phase [22]. This study further implies the requirement of some developmental signals: one signal activating SRY and launching the initiation phase, and two signals defining the transition from the initiation to the maintenance phases by, on the one hand, inhibiting the WNT4 pathway, and activating FOXL2 on the other hand.

Here, a similar approach is proposed with the dynamical analysis of the gene network controlling primary sex determination in the chicken. Relying on experimental data and prevailing scenarios, we constructed the regulatory network encompassing the main genes involved in the process. The resulting logical model complements current experimental work, assessing the Z-dosage and dominant W-chromosome hypotheses, and underscoring the global dynamics that underlies chicken sex determination.

## 2. Methods

The proposed model was defined relying on the logical formalism [23,24], and using the software tool GINsim [25]. Further details on this modelling framework are provided in the electronic supplementary material.

A gene regulatory network is represented by a directed graph in which nodes and arcs stand for genes (or regulatory component) and interactions, respectively. Each node is assigned a discrete variable that describes the component state, its maximal value defining the highest qualitative functional level (this maximal level is 1 in the simplest, Boolean case). Multilevel variables are used when distinct functional concentrations of a regulatory product need to be considered. Each arc embodies a regulatory interaction and is assigned a threshold, which defines the smallest functional level of the regulator, source of the interaction, for which the interaction is operative (value 1 by default). Logical functions qualitatively describe the effects of the interactions controlling the component levels.

The model dynamics is defined according to a specific updating scheme, here the asynchronous update. This scheme specifies that when several components are called to change their levels (due to the effects of their regulators), updates are performed independently, possibly leading to concurrent transitions and thus alternate trajectories [26]. The asynchronous dynamics can be refined by considering priority classes, which specify qualitative restrictions regarding rates at which components change their levels, thus resolving some concurrent updates. Properties of interest relate to the model attractors and their reachability from relevant initial conditions. These model attractors correspond to cell phenotypes (here male or female differentiated gonads).

Finally, a thorough understanding of a genetic network controlling a biological process requires a dynamical analysis not only under wild-type conditions, but also under mutant conditions. In a logical model, mutations are easily defined by simply restricting the values of the corresponding variables (a higher value for a gain-of-function, a null value for a loss-of-function).

Model files are available at http://ginsim.org/model/sex_determination_chicken and the GINsim software can be freely downloaded at http://ginsim.org.

## 3. Results and discussion

### 3.1. Model definition

Relying on data obtained from experiments in chickens, we assembled the gene regulatory network (electronic supplementary material, figure S1). It includes current hypotheses for chicken sex determination, namely the Z-dosage and the dominant W hypotheses (see Introduction). Indeed, these scenarios need not to be exclusive as demonstrated by ZZW intersexes [11,12], which could result from antagonistic functions of male Z-linked and female W-linked genes. Alternatively, a female W-linked gene could exert its function by inhibiting/modulating the Z-linked DMRT1 gene. In this respect, the most appealing proposal argues for such a W-linked gene controlling the transcriptional state of the hypermethylated region (MHM) of the Z chromosome nearby DMRT1 locus [27]. Briefly, this W-linked gene would encode for a factor causing hypomethylation of the MHM region. It would be transcriptionally active, producing non-coding RNAs coating this region of the Z chromosome, thus preventing gene transcription and reducing the transcription of closely located genes, such as DMRT1. In ZZ individuals, the MHM region remains hypermethylated (transcriptionally inactive) so that DMRT1 is fully expressed. Accordingly, the proposed network includes three input components to convey the chromosomal constitution of the gonad: Z1 and Z2 embody identical Z-chromosomes (carrying DMRT1), whereas W stands for a W-chromosome (carrying the putative gene whose product modulates DMRT1 expression level).

**Figure 1.**
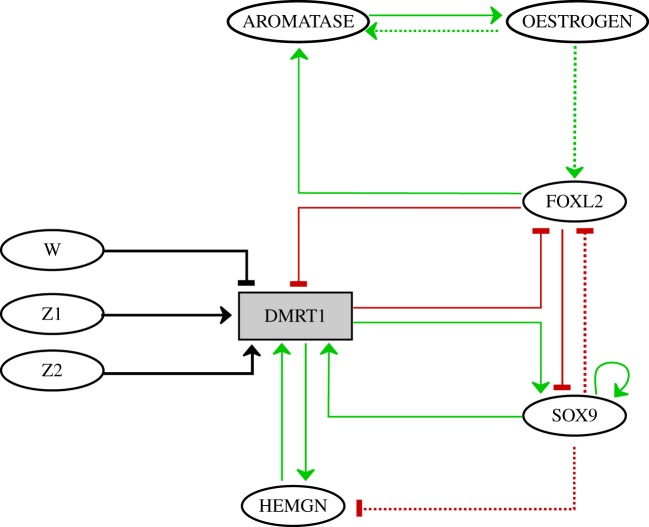
The (simplified) gene regulatory network controlling chicken primary sex determination (see electronic supplementary material, figure S1 for the complete network). Normal green and blunt red arrows represent positive and negative interactions, respectively, and dashed arrows indicate indirect or proposed interactions. Z1 and Z2 represent each a Z chromosome, whereas W represents a W chromosome; the sex chromosome constitution (ZW or ZZ) is thus specified by an adequate combination of these input components.

For the sake of simplicity, we reduced the extended network of electronic supplementary material, figure S1, resorting to an appropriate reduction method that preserves essential features of model behaviours [28] (see the electronic supplementary material). The analyses were performed on the resulting network shown in figure 1, which encompasses the core interactions that control the sexual development of the bi-potential gonad. The set of experimental results backing the gene interactions of this sub-network follows.

#### 3.1.1. Z1, Z2→DMRT1

DMRT1 is the best candidate for a primary sex determination signal. First, it is located in the Z chromosome and has no homologue in the W chromosome so that DMRT1 is present in a double dose in males (ZZ) and in a single dose in females (ZW). Second, DMRT1 is specifically expressed in the gonads of chicken embryos, a higher expression in males than in females [3,4]. Third, it is expressed in female-to-male sexual reversed ZW embryos [5]. Finally, elimination of DMRT1 function in ZZ males causes male-to-female sex reversal [6]. For methodological simplicity, it was considered that the Z chromosome ‘would act as if it was a DMRT1 activator’; hence the arrows from Z1 and Z2 onto DMRT1.

#### 3.1.2. DMRT1→HEMGN and HEMGN→DMRT1 interactions

Expression of DMRT1 starts at day 3.5, while expression of HEMGN begins at day 5.5 after incubation [29]. HEMGN expression is induced in female ZW gonads under overexpression of DMRT1 [30]. Overexpression of HEMGN in female ZW gonads causes elevation of both DMRT1 and SOX9 expression [29]. These embryos show high levels of HEMGN protein throughout the gonad, yet SOX9 protein is found only in the region of the gonad where DMRT1 is also expressed [5,29]. Moreover, putative HEMGN-binding sites have not been reported in the chicken SOX9 promoter [31]. Here, we propose that HEMGN does not interact directly with SOX9 and that HEMGN participates in the upregulation of the DMRT1, which in turn activates SOX9, which then feedbacks onto DMRT1 to maintain its high expression.

#### 3.1.3. DMRT1→SOX9 interaction

Expression of SOX9 starts at day 6.5 and DMRT1 starts being expressed at day 3.5 [4]. Elimination of DMRT1 activity down-regulates SOX9 expression [6]. SOX9 expression is induced in female ZW gonads under overexpression of DMRT1 [30]. A putative DMRT1 consensus-binding site within the promoter of chicken SOX9 has been identified that overlaps with the SOX9-binding site [31].

#### 3.1.4. SOX9→DMRT1 interaction

HEMGN expression starts to disappear after day 8.5 [29]. Then, how is DMRT1 expression maintained? Here, it is considered that once SOX9 is activated, a positive feedback loop between DMRT1 and SOX9 is set up, locking the expression of both.

#### 3.1.5. SOX9--|HEMGN interaction

HEMGN is mainly expressed in male ZZ gonads from day 5.5 onwards, showing an increased expression with a peak at day 8.5, and co-localizing with SOX9 expression [29]. HEMGN expression is then lost [29], coinciding with SOX9 upregulation by day 8.5 [32,33]. Here, we assumed that SOX9 participates, directly or indirectly, to HEMGN repression after its peak of expression. For simplicity, a direct negative interaction from SOX9 upon HEMGN was thus considered (figure 1).

#### 3.1.6. SOX9→SOX9 interaction

The chicken SOX9 promoter contains a SOX9-binding site (previously identified in SOX9 auto-regulation in mice [5]), suggesting that SOX9 is auto-regulated [31].

#### 3.1.7. DMRT1--|FOXL2 and FOXL2--|DMRT1 interactions

Elimination of DMRT1 activity in male chicken gonads leads to an ectopic expression of FOXL2 and of AROMATASE, the latter being a consequence of the former [6]. Overexpression of AROMATASE in the male gonad determines its ovarian development, showing an activation of FOXL2 and a repression of DMRT1 [34].

#### 3.1.8. FOXL2--|SOX9 interaction

SOX9 promoter contains a FOXL2-binding site (overlapping with SOX9- and DMRT1-binding sites) [31].

#### 3.1.9. FOXL2→AROMATASE interaction

FOXL2-binding sites have been found in the promoter of AROMATASE [35], and FOXL2 can activate the AROMATASE promoter *in vitro* [36–38].

#### 3.1.10. AROMATASE→OESTROGEN interaction

AROMATASE is the terminal enzyme for OESTROGEN synthesis [39].

#### 3.1.11. OESTROGEN→FOXL2 interaction

FOXL2 is an activator of AROMATASE (see above), and its expression is elevated in male gonads after AROMATASE expression, suggesting a feedback mechanism between FOXL2 and AROMATASE [34]. This feedback does not seem to be directly mediated by OESTROGEN because no OESTROGEN response elements have been detected in FOXL2 promoter [40]. However, assuming that this feedback can be mediated by an OESTROGEN-regulated gene, we included a positive interaction from OESTROGEN to FOXL2.

For the sake of parsimony, the genes (and their products) were assumed to have single functional levels (i.e. the associated variables are Boolean), except for the gene DMRT1. This was associated with a multilevel variable accounting for two distinct functional levels (i.e. it can take values 0, 1 and 2), following the Z-dosage hypothesis. The equations defining the functional levels of the model components are presented in electronic supplementary material, table S1.

The proposed model rests on a set of features substantiated by the experimental evidence listed above: (i) the sexual fate of the gonad is assumed to rely on two (compatible) scenarios for which either initial amounts of DMRT1 product in ZZ are higher than in ZW gonads (due to higher doses of this Z-linked gene, and to the lack of dosage compensation), or DRMT1 initial amounts are similar in both gonads, but promptly decrease in ZW gonads due to the function of a W-linked gene; (ii) HEMGN is assumed to induce DMRT1 upregulation; (iii) SOX9 is assumed to regulate itself and to repress HEMGN function; (iv) the proposed cross-inhibition between DMRT1 and FOXL2 determines their final functional levels; (v) the sexual development engaged by the bi-potential gonad depends on SOX9 final state: if SOX9 remains active, male development ensues, otherwise female development follows; and (vi) in female development, hormones, basically OESTROGEN, control FOXL2 function.

### 3.2. Model simulations

#### 3.2.1. General settings

Because ZZ and ZW gonads are originally identical, the initial states selected to perform the simulations differed only in the input node levels: for ZZ gonads, Z1 and Z2 were both set to 1 and W is set to 0, whereas for ZW gonads, Z1 and W were both set to 1 (Z2 being 0). Unless otherwise specified, for ZZ and ZW gonads, the DMRT1 initial level was set to its intermediate level (1), and all remaining components were set to their lower level (0).

Simulations, performed under an asynchronous updating combined with priorities when needed, emulate the developmental dynamics of the undifferentiated gonad into either testis or ovary. Updating priorities revealed putative temporal restrictions ensuring an appropriate sexual development of the gonad.

We considered the following criteria for defining the sexual phenotypes: expression of DMRT1 and SOX9 and absence of FOXL2, AROMATASE and OESTROGEN indicate testis identity, while expression of FOXL2, AROMATASE and OESTROGEN and absence of DMRT1 and SOX9 indicate ovary identity.

#### 3.2.2. Wild-type gonads

For the wild-type ZZ gonads, the simulation led to two stable states: one corresponding to the ovary identity and the second to the testis identity. This indicated that, under an unrestricted asynchronous update, the model could reproduce both male and female developmental pathways. A closer examination of the dynamics allowed identification of constraints (i.e. priority classes) ensuring the reachability of the sole male phenotype from a ZZ gonad initial state (see electronic supplementary material, figure S2): activation of DMRT1 and HEMGN (i.e. level increases) should be faster than FOXL2 activation. This priority setting is denoted PC1 (table 1).

**Table 1.** Description of the update settings used in the simulations of the wild-type and mutant gonads (table 2); see the electronic supplementary material for details on priorities.

name	description—comments
no priorities	all events are considered asynchronously
PC1	DMRT1 & HEMGN increases are faster than FOXL2 increase
PC2	DMRT1 decrease is faster than HEMGN & DRMT1 increases
PC3	DMRT1 decrease is slower than HEMGN & DRMT1 increases, which are faster than FOXL2 increase (i.e. W-linked effect overcome, in addition to PC1 setting)
PC4	FOXL2 increase is faster than HEMGN & DMRT1 increases (counterpart of PC1, could be explained by the presence of some FOXL2 activator)

For the wild-type ZW gonad, starting with a DRMT1 initial level at 0, the model simulation produced a single stable state corresponding to the ovary identity (i.e. in this case, a W-linked gene, here represented by the input node W, inhibiting DRMT1 would be dispensable). However, when starting with an intermediate level of DMRT1, the simulation led to two stable states, corresponding to the ovary and testis identities. To ensure the reachability of the sole female phenotype, this initial DMRT1 should promptly decrease (due to the inhibitory effect of the putative W-linked gene), faster than e.g. HEMGN increase. Then having the DRMT1 level at 0, the simulation would proceed towards the female phenotype as previously (see electronic supplementary material, figure S2). This led to the definition of the priority setting referred to as PC2: when DMRT1 inhibition can occur, it should be faster than any other event.

**Figure 2.**
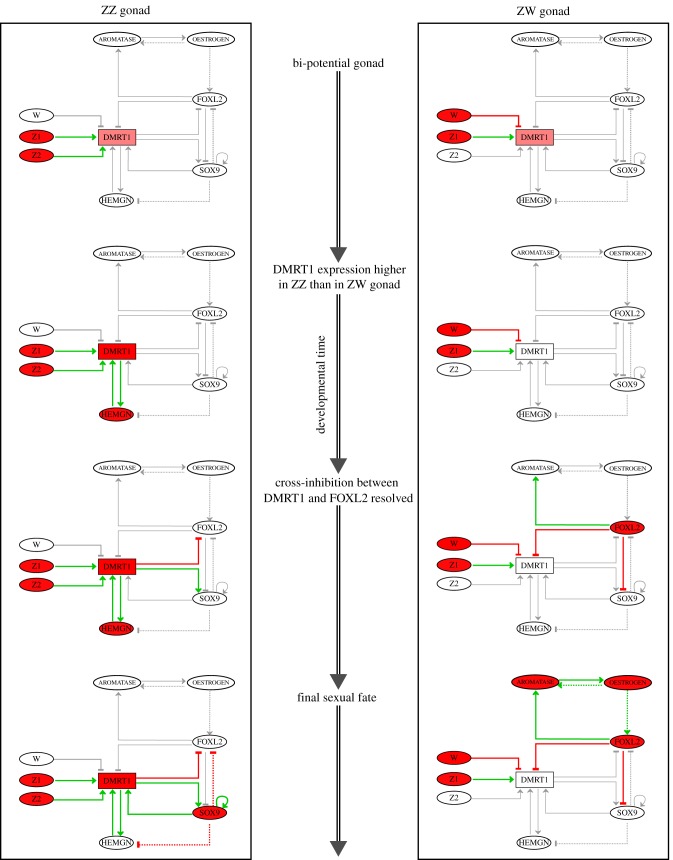
Dynamics of the gene regulatory network for the ZZ and ZW chicken bi-potential gonads. Coloured (respectively, grey) arrows indicate operative (respectively, non-operative) interactions; components in red are at their maximal levels (pink for the intermediate level of DMRT1), white components are at their minimal level 0.

*Development of the ZZ bi-potential gonad* (figure 2). At the beginning of differentiation, the amount of DMRT1 is stabilized (due to the absence of a W-linked gene effect) and thus is sufficient (value 1) to activate HEMGN. This in turns feeds back onto DMRT1 causing its upregulation to a higher functional level. As a consequence, DMRT1 activates SOX9, which is then able to maintain its activity and to repress HEMGN. Finally, SOX9 expression allows DMRT1 to maintain its high functional level that blocks FOXL2 expression. Consequently, DMRT1 is permanently activated throughout development due to SOX9 auto-regulation, thus leading to the male development of the ZZ gonad.

*Development of the ZW bi-potential gonad* (figure 2). At the beginning of the differentiation process, either DMRT1 initial level is low (value 0) in a Z-dosage scenario alone, or it is intermediate and lowered by the action of the W-linked gene, so that DMRT1 cannot activate HEMGN. As a consequence, DMRT1 does not reach the high level required to activate SOX9. Meanwhile, FOXL2 reaches its functional level (value 1), which is maintained through positive feedback including AROMATASE and OESTROGEN. Thus DMRT1 and SOX9 remain inactive, whereas maintained activities of FOXL2, AROMATASE and OESTROGEN elicit the female development of the ZW gonad.

Summarizing, the sexual fate acquired by the bi-potential gonad results from the resolution of the cross-inhibition between DMRT1 and FOXL2—at the initial phase of differentiation—that determines whether SOX9 auto-regulation is implemented or prevented. This resolution is driven by the initial amount of DMRT1 as controlled by the gonad chromosomal constitution. Temporal restrictions suggest that: (i) the initial FOXL2 basal activation is slower than the upregulation of the male genes HEMGN and DMRT1 and; (ii) the inhibition of DRMT1 by a putative W-linked gene must occur straightaway at the beginning of the differentiation process.

#### 3.2.3. Mutant gonads

A series of perturbations of the model were simulated in the form of loss-of-function (LOF) and gain-of-function (GOF) mutations. Initial states were set as for the wild-type gonads, with an intermediate level of DRMT1 and a level compatible with the perturbed component (i.e. 1 for the gain-of-function of a Boolean node). In some cases, temporal restrictions needed to be considered, providing insights into the dynamics of involved mechanisms. Priority settings are described in table 1. However, unless otherwise specified, the simulations were performed under an asynchronous updating. The results, described below and summarized in table 2, match existing experimental observations, otherwise they provide predictions that remain to be tested.

(1) Simulation of a DMRT1 LOF ZZ gonad led to an ovary phenotype, whereas a DMRT1 full GOF ZW gonad (DMRT1 restricted to level 2) led to a testis phenotype, in agreement with experimental results [6]. Moreover, a partial DMRT1 GOF ZW gonad (level restricted to 1) led to an ovary phenotype, showing that the initial amount of DMRT1 product is critical to drive the sexual fate of the bi-potential gonad.
(2) Simulation of a HEMGN LOF ZZ gonad led to an ovary phenotype, whereas a HEMGN GOF ZW gonad led to a testis phenotype, in agreement with experimental results [29]. For the latter, PC1 priority setting needed to be considered, suggesting that FOXL2 activation should be slower than HEMGN and DRMT1 activation. Altogether, these results point to a role of HEMGN in DMRT1 upregulation.
(3) Simulation of a SOX9 LOF ZZ gonad, considering PC1 priority setting (similar to the one selected for the wild-type situation), led to neither male nor female fates, considering that both DMRT1 and SOX9 products are required for testis development. Note that, because HEMGN is not repressed, DMRT1 expression is maintained at its highest level (value 2). In other words, the model predicted the maintenance of HEMGN expression in ZZ gonads lacking SOX9 function. Elevated expression of DMRT1 would prevent FOXL2 expression and thus AROMATASE and OESTROGEN expression, avoiding ovary development. This model prediction for ZZ gonads lacking SOX9 function relies on the assumption that SOX9 participates to HEMGN repression (for details, see SOX9-HEMGN interaction). However, simulation of a ZZ gonad with double LOF of SOX9 and HEMGN led to an ovary phenotype. In this setting, the model predicts that the ovary genes FOXL2, AROMATASE and OESTROGEN can be expressed because DMRT1 expression cannot be maintained. Finally, simulation of a SOX9 GOF ZW gonad resulted in a testis phenotype.
(4) Simulation of a FOXL2 LOF ZW gonad led to a testis phenotype, provided PC3 priority setting (table 1). That is to say, DMRT1 intermediate initial level (value 1) should not decrease before HEMGN activation (see discussion below). Furthermore, the model predicted an ovary development of a FOXL2 GOF ZZ gonad.
(5) Simulation of an AROMATASE LOF ZW gonad led to a testis phenotype, in agreement with experimental results [41,42]. Reaching this phenotype required the consideration of PC3 priority setting (see discussion below). Simulation of a ZZ gonad with a GOF of AROMATASE led to an ovary phenotype, also in agreement with experimental results [34]. However, reaching this female phenotype now required a fast FOXL2 activation (PC4 priority setting) or an initial FOXL2 expression (see discussion below).
(6) Simulation of a ZW gonad with a LOF of OESTROGEN led to a testis phenotype, while simulation of an OESTROGEN GOF ZZ gonad resulted into an ovary phenotype. Both results agree with experimental observations [43,44]. In both cases, reaching the correct phenotype required temporal restrictions regarding DMRT1 and FOXL2 similar to those defined for AROMATASE perturbations (see below).

**Table 2.** Stable states reached by the gene network and the corresponding phenotypes (testis, ovary) under wild-type and mutant conditions. Update settings are defined in table 1: asynchronous update with no priorities or priority settings ensuring the reachability of a unique phenotype (see text). (Online version in colour.)

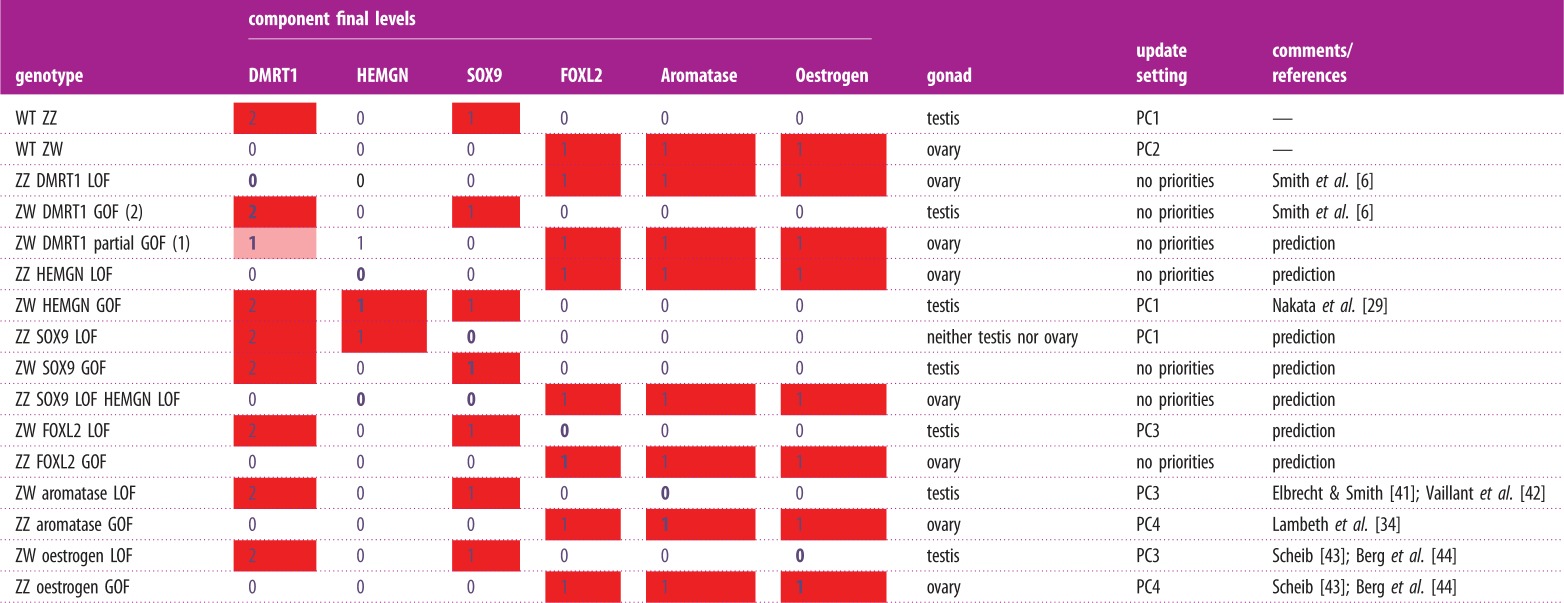

To reproduce female-to-male sex reversal of ZW gonads carrying loss-of-function mutations at the female-promoting genes (FOXL2, AROMATASE and OESTROGEN), we had to start the simulations with an intermediate level of DMRT1 and to apply PC1 priority setting. This ensures that, in the absence of proper activators, FOXL2 increase is slower than HEMGN and DMRT1 upregulation (as in the case of the wild-type ZZ gonad). In contrast, for simulations of ZZ gonads carrying gain-of-function mutations of AROMATASE or OESTROGEN, male-to-female sex reversal could be achieved provided the consideration of PC4 priority setting (FOXL2 increase faster than that of HEMGN and DMRT1). This could be justified by the overexpression of FOXL2 activators.

Altogether, the temporal restrictions on DMRT1 or FOXL2 discussed above point to the chief role of the resolution of the cross-inhibition between these two genes over the initial phase of sexual differentiation. Simulations of mutant gonads in which DMRT1 and FOXL2 regulators are perturbed enabled the assessment of how the interplay of their activators and repressors drives the functional levels of these two genes. The sexual fate of the gonad is implemented during a developmental time window characterized by the successful or failed set-up of SOX9 auto-regulation, this decision being controlled in a cell autonomous manner as shown in the case of gynandromorph chickens [45].

**Figure 3.**
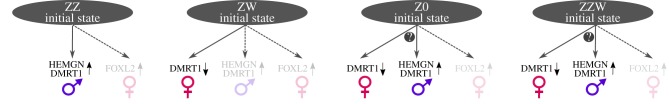
Illustration of the concurrent events leading to distinct sexual fates for different chromosomal constitutions of the gonad. Priority settings to settle these conflicting events are proposed for the ZZ and ZW gonads (table 1). (Online version in colour.)

Our model further allowed us to explore the role of a W-factor in triggering the sexual fate of the chicken gonad. In this respect, we simulated Z0 (a single Z and no W chromosome) and ZZW gonads, and figure 3 illustrates the concurrent events involved, depending on the chromosomal composition of the gonad.

Considering an intermediate initial level of DMRT1 like the wild-type initial state, the asynchronous simulation of a Z0 gonad led to two stable states, corresponding to the ovary and testis identities (figure 3). Two concurrent events at the initial state produce this multi-stability, similar to the ZW wild-type case: the decrease in DMRT1 level that is due here to the absence of the second Z chromosome (and not to the presence of the W-linked gene), and the increase in HEMGN (because of DMRT1 intermediate level). The simulation of the ZZW gonad also led to both ovary and testis phenotypes. In this case, the concurrent events are the same, but are due to different effects: the decrease in DMRT1 level is caused by the W-linked gene, whereas the HEMGN increase is due to DMRT1 intermediate level, maintained thanks to the high dose of this Z-linked gene.

The priority settings (PC1 and PC2) inferred for the wild-type simulations are not necessarily valid for the Z0 and ZZW gonads. Indeed, as mentioned above, the mechanisms associated with the concurrent events differ, in particular concerning DMRT1 decrease. As illustrated in figure 3, the model demonstrated the multi-stability potential, i.e. the possible reachability of both sexual fates from the initial state. The final fate results from the settlement between the decrease in DMRT1 level (female fate) and its maintenance, leading to an increase in HEMGN level and thus a further increase in DMRT1 level (male fate).

Collectively, these results strongly support the view of a female-role played by a putative W-linked gene in chicken gonadal sex determination. It is argued here that this W-factor would operate as a secondary device to strengthen the difference of the amounts of DMRT1 product in ZW and in ZZ gonads at the initiation step of sexual development.

## 4. Concluding remarks

The modelling approach presented here aimed to decipher primary sex determination in the chicken. We relied on experimental data and made a few assumptions (see the electronic supplementary material for details) to define a logical model with the following features:

(1) The model integrates current hypotheses for chicken sex determination; namely the Z-dosage and the dominant W hypotheses. The Z-linked DMRT1 is widely considered as the potential primary sex determination signal (Z-dosage hypothesis), so that the different initial amounts of DMRT1 in ZZ and ZW gonads ultimately determine the development of testes and ovaries. The W-factor would function at the initiation step as a secondary device, through DMRT1 inhibition, reinforcing the reduced amount of DMRT1 product in ZW versus ZZ gonads.
(2) The final sexual fate of the bi-potential gonad results from the resolution of the mutual negative interaction between DMRT1 and FOXL2. Our model suggested that some developmental constraints are instrumental to this resolution process. These constraints correspond to qualitative restrictions (priority classes in table 1) regarding the relative rates at which DMRT1, FOXL2 and HEMGN change their functional levels.
(3) HEMGN plays a role in the resolution of DMRT1-FOXL2 mutual negative interaction in favour of DMRT1 by boosting its initial expression in ZZ gonads.
(4) The proposed SOX9 auto-regulation is a determinant for the male pathway through the positive effect of SOX9 upon maintenance of DMRT1 expression for testis development.
(5) In chickens, in contrast with what happens in mammals, OESTROGEN plays a role in primary sex determination by means of its positive effect on the maintenance of FOXL2 function during ovary development.

The proposed model has two key elements. The first concerns the formation of the primary signal that triggers the sexual development of the chicken gonad. This signal has been defined by integrating both the Z-dosage and dominant W hypotheses: the Z-linked DMRT1 gene and the putative W-factor acting upon the Z-linked MHM region. The second key constituent of the model is the mutual inhibition between DMRT1 and FOXL2, which ensures the maintenance of the chosen sexual pathway. An essential feature of our model is that the primary signal is responsible for resolving this cross-inhibition.

Can this model of chicken sex determination be extended to all birds? In this regard, it is pertinent to consider the following results related to the formation of the primary signal.

On the one hand, the W-linked genes HINTW (also known as WPKCI), FAF and FET1 show an expression level unaltered after masculinization of ZW gonads as demonstrated by Hirts *et al.* [46] who cloned and characterized the homologues of these genes. This suggests that these genes play no role in gonadal sex determination neither in zebra finch nor in emu, as previously reported in the chicken for HINTW and FET1 (see Introduction). Further identified in this study is a discrepancy regarding HEMGN, which does not show the male-specific upregulation observed in chickens [46]. The authors propose that ‘The Z-linked DMRT1, and not the W sex chromosome, regulates gonadal sex differentiation in birds.’ On the other hand, the MHM region is conserved across the Galloanserae clade to which chickens belongs, but is absent from zebra finch (Neovaes clade) and in emu (Paleognathae clade) as demonstrated by Wright *et al.* (2015) by characterizing this region in a large portion of the avian lineage [47].

Altogether, these results indicate that our model cannot be straightforwardly extended to non-Galloanserae birds. However, the results of Hirst *et al.* [46] and Wright *et al.* [47] do not unambiguously exclude a role of the W chromosome in avian sex determination, as evidenced by reported ZZW females that produce offspring in natural populations of the great reed-warbler (*Acrocephalus arundinaceus*) [48] and of the plover (*Charadrius alexandrinus*) [49]. These two birds belong to the Neovaes clade, which is distantly related to the chicken lineage (Galloanserae clade). Indeed, our model can account for these ZZW females. It would suffice to assume that the proposed inhibition of DMRT1 by the W factor is stronger in these ZZW birds than in the chicken, so that the lack (or very low amount) of DMRT1 product would tilt the DMRT1-FOXL2 balance in favour of FOXL2, leading to subsequent female development. Thus, it may well be that the role of HEMGN and/or of the W-factor/MHM region in the chicken is handled by other genes in other birds. However, we cannot discard that the primary sex determination signal in the chicken, as proposed here, might represent an evolutionary novelty that arose in the chicken lineage after its separation from the other avian clades. This would resemble the case of higher vertebrates, where the gene SRY arose in the Theria lineage after its separation from the Prototheria lineage. Nevertheless, we believe that our model sheds light on how the primary signal of whatsoever nature triggers the sexual programme of the gonad through the resolution of the mutual inhibition of DMRT1 and FOXL2, two genes conserved across birds.

Returning to the chicken—nowadays the most feasible experimental model for studying sex determination in bird—a key question remains open; namely what is the nature of the putative W-factor? Following our model—based on Teranishi *et al.* [27] observation that the methylation degree of the Z-linked MHM region depends on the presence of a W-chromosome—the W-factor could be either a specific demethylase or a methylase inhibitor acting on the MHM region. In this respect, a bioinformatic search of putative W-linked demethylases and/or methylase inhibitors in the chicken genome may provide relevant candidates. Transcriptomic analyses of early male and female gonads may constitute another valuable source of information because, according to our model, the W-factor would act at the initial step of gonadal sexual development. Additionally, checking for a potential sex reversal, treatment of early ZZ embryos with demethylases or methylase inhibitors could be a relevant test whose feasibility remains to be decided by experimentalists.

Finally, comparing primary sex determination in mammals and chickens, one can formulate three observations that are relevant from the evolutionary point of view. First, the mutual repression of DMRT1 and FOXL2 in mammals is already present in chickens although its biological function differs in the two vertebrates. In the chicken, the DMRT1-FOXL2 interaction is involved in the initiation and maintenance of the gonadal sexual development, whereas it is only involved in the maintenance state in mammals. Indeed, DMRT1 functions as a primary signal in chickens, which is not the case in mammals where this signal is provided by SRY, a gene acquired during the mammal evolutionary lineage. Second, the auto-regulation of SOX9 observed in mammals would be already present in chickens. Moreover, SOX9 would play the same role in both vertebrates. Third, HEMGN in chickens would overcome FOXL2 inhibition by increasing DMRT1 expression, similarly to SRY in mammals, which was proposed to overcome ß-CATENIN inhibition by boosting SOX9 expression [22]. In both cases, the set-up of SOX9 auto-regulation is crucial for male development so that, in case of a failure, female development ensues (figure 2).

## Supplementary Material

Supplementary text

## Supplementary Material

Figure S1

## Supplementary Material

Figure S2

## Supplementary Material

Table S1

## Data accessibility

Model files are available at http://ginsim.org/model/sex_determination_chicken.
