# Supplementary material for: Logical modelling uncovers developmental constraints for primary sex determination of chicken gonads
Source: J R Soc Interface. 2018 May 23;15(142):20180165. doi: 10.1098/rsif.2018.0165 (PMC6000168; doi:10.1098/rsif.2018.0165)
Supplement: Supplementary text [file rsif20180165supp1.pdf]

Journal of The Royal Society Interface

Logical modelling uncovers developmental constraints for primary sex determination of chicken gonads

Lucas Sánchez[1] and Claudine Chaouiya[2]

1. Centro de Investigaciones Biológicas (C. S. I. C.), Madrid, Spain.

2. Instituto Gulbenkian de Ciência – IGC, Oeiras, Portugal

Supplementary text

Content

1. **Logical modelling framework** 1
   1.1. **Basics** 1
   1.2. **Simulation of genetic perturbations** 2
   1.3. **Updating schemes and Hierarchical Transition Graphs** 2
2. **Gene network controlling primary sex determination in chicken** 3

1. Logical modelling framework

1.1. Basics

The logical formalism proved well adapted to model genetic networks for which no detailed quantitative date is available [1,2,3]. In this framework, a regulatory network is represented in terms of a directed graph. Each node (gene or regulatory product) is assigned a discrete variable with a maximal level, which defines the highest qualitative functional level of that node (this maximal level is 1 in the simplest Boolean case). Whenever distinct functional concentration of a regulatory product needs to be considered, multilevel variables are used. Each arc embodies a regulatory interaction and is assigned a threshold, which defines the smallest functional level of the regulator (source of the interaction) from which the interaction is operative. Logical functions qualitatively describe the effects of each interaction or combination of interactions controlling the states of the network nodes. The logical functions for the network model of Figure 1 are given in the Supplementary Table S1.

A state of the network is represented by a vector, which encompasses the current (discrete) levels of the nodes. Given a state, one can determine which interactions are operative and the values of the logical functions indicate the nodes called to change their levels (i.e. those nodes whose current levels differ from the values of the logical functions). In general, for a given state, all possible elementary transitions (*i.e.* switching of a single node level to a neighbouring integer level) are considered, thus leading to as many outgoing transitions as updating calls (asynchronous updating); these transitions correspond to concurrent events. The dynamics is thus defined by the regulatory graph and associated logical functions, encompassing a finite number of, possibly concurrent, dynamical pathways. These are in turn represented in the form of a State Transition Graph (STG), in which the nodes represent the network states and the (directed) edges represent the transitions between states. Section 1.3 below provides further details on the updating schemes and the representation of the dynamics in terms of graphs.

The software tool GINsim [4] was used for the simulation and logical analysis of the model, which is provided as a Supplementary File, in SBML qual-format [5]. Model files are further available at http://ginsim.org/model/sex_determination_chicken, and the GINsim software can be freely downloaded at http://ginsim.org.

1.2. Simulation of genetic perturbations

Within the logical framework, simulation of genetic perturbations is straightforward. A loss-of-function mutation of a given gene implies that this gene produces a non-functional product (or no product at all), which amounts to assign the value zero to the corresponding variable and function. In contrast, the ectopic expression of a gene implies that this gene is expressed in an unregulated manner beyond its normal spatio-temporal expression domain. This can be accomplished by forcing the corresponding variable to take higher values (for detail of this formal treatment of mutations, see [6]).

1.3.Updating schemes and Hierarchical Transition Graphs

Model dynamics are defined by specifying some initial state(s) and an updating scheme, which refers to how node levels are updated. As previously mentioned (Section 1.1), the asynchronous update is generally chosen as it amounts to generate all possible trajectories from the specified initial conditions: e.g. when two node levels are changed,

these updates are done asynchronously, defining two concurrent transitions towards alternative successor states. The resulting STG may then include bifurcation states from which, depending on the choice of the successor state, the final stable state may differ; in other words, in the absence of any further constraint, two alternative phenotypes are reachable.

For our model of the genetic control of primary sex determination in chicken, constraints on concurrent updates (transitions) needed to be considered to get a unique stable state matching the expected phenotype (testis or ovary). Such constraints are expressed in terms of priority settings, *i.e.* temporal orders between node updates [7]. To uncover these priorities, we relied on a compact representation of the dynamics, called Hierarchical Transition Graphs (HTGs) (Figure S2).

We briefly introduce HTGs, which provide compact representations revealing attractors and their basins of attraction, as well as transient oscillatory behaviours [8].

Let us first recall that a Strongly Connected Component (SCC) is defined as a maximal strongly connected subgraph (i.e., a maximal subset of states in the STG, such that there is a path connecting each state to any other state). These SCCs are termed *trivial* when they are reduced to a unique state, *complex* otherwise. To construct a HTG, states of the STG are gathered into a single node if:

- they belong to a transient complex SCC, or
- they belong to an attractor i.e., a trivial or a complex SCCs with no outgoing transitions, or
- they define trivial SCCs from which the same complex and terminal SCCs are reachable.

Nodes of a HTG are thus defined as sets of states. The arcs between these nodes denote the existence of (at least) a transition between two states of the corresponding sets of states. They can be labelled by the associated updates (see Figure S2).

2. Gene network controlling primary sex determination in chicken

The gene network that controls primary sex determination in chicken is shown in Figure S1. This network has been fundamentally constructed from data gathered from experiments on

chicken, which is the experimental model for avian sex determination. Similar to the analysis of the gene network controlling the sexual development of the bi-potential gonad in mammals [9], a set of reductions were performed that do not affect the main biological features of the regulatory network [10]. These reductions led to the sub-network shown in Figure 1, on which the analysis was carried out. In what follows, the justification of the genes and their interactions not included in the simplified version are presented:

1. It is considered that SF1 does not interact with SOX9 as no SF1-binding sites have been identified in the chicken promoter of gene SOX9 [11].

2. The expression of AMH is induced in female ZW gonads after over-expression of DMRT1 [12]. It is considered here that this positive effect of DMRT1 is indirect via SOX9.

3. SF1 alone cannot activate AROMA in chicken ovaries, as expression of the former begins at day 3.5 and expression of the latter at day 6.5 of incubation [13]. The additional gene could be FOXL2 (its product binds to the AROMA promoter) whose expression precedes that of AROMA [14].

4. AMH is expressed in both male and female embryos —although at very low levels in female compared to male embryos— a full day before SOX9 expression is initiated in males [15-17]. Thus, it seems that SOX9 is not required for the initiation of AMH expression but for the up-regulation observed in males. It has been proposed that SF1 would determine the initial expression of AMH in both sexes, whereas the male-specific up-regulation of AMH would depend on both SF1 and SOX9 [17]. This proposal was followed to construct Figure S1.

5. The activation of SF1 by OESTROGEN was based on the observation that SF1 was up regulated in the gonads of male-to-female sex reversal ZZ embryos exposed to OESTROGEN during the period of sex determination [18].

6. AROMATASE activation by SF1 was based on the existence of a putative binding site for SF1 in AROMATASE promoter region [19,20]. SF1 is expressed in the male and female chicken undifferentiated gonad, and after the onset of gonadal sex differentiation SF1 and AROMATASE genes are up-regulated at the same time, between days 6 and 7 in the female gonads [15,16]. However, SF1 alone cannot activate AROMATASE in ovaries, as expression of the former starts at day 3.5 and expression of the latter at day 6.5 of incubation [21]. The additional gene could be FOXL2 whose expression precedes that of AROMATASE [14].

7. The activation of AMH by SF1 was based on the existence of a putative binding site for SF1 in the promoter region of AMH gene [17]. SF1 is expressed in the undifferentiated gonads of both sexes prior to AMH expression and remains expressed during gonadal sex differentiation when AMH expression increases in male gonads [15,21,22]. SF1 also remains expressed in gonads of sex-reversed females [23].

8. The Amh promoter contains a potential OESTROGEN response element [17]. Since OESTROGEN and AMH are antagonists, it is considered that the interaction of OESTROGEN upon AMH is negative.

9. The expression of AMH is induced in female ZW gonads after over-expression of DMRT1 [12]. Since the AMH promoter contains putative response elements for SOX9 [17], it is considered here that the positive effect of DMRT1 on AROMATASE is indirect via SOX9.

10. Over-expression of AMH inhibits the expression of FOXL2 and AROMATASE [24]. Since FOXL2 is required for AROMATASE expression, it is considered that the effect of AMH upon AROMATASE is indirect through its effect on FOXL2.

11. FGF9, which is initially expressed in the bi-potential gonad of both sexes, is differently expressed when the gonads start to develop sex-dependently: FGF9 expression is higher in male ZZ than in female ZW gonads [25]. Over-expression of FGF9 causes an expansion of the male and females gonads by increased cell proliferation, with DMRT1 and SOX9 expressed in the expanded region of male but not of females gonads [25]. Hence, it was considered here that FGF9 does not participate in SOX9 activation but in its maintenance like in mammals, where SRY activates SOX9 [26,27]. In the chicken, the activation of SOX9 would depend on the expression level of DMRT1.

12. WNT4 is expressed in the bi-potential gonads of both sexes, and it is later up-regulated in female gonads [28]. That is, both WNT4 and FGF9 have a similar initial expression but show later opposite expressions: WNT4 is higher in female gonads, whereas FGF9 is higher in male gonads. Like in mammals, the chicken undifferentiated gonad can develop into either testis or ovary. The sexual developmental plasticity of the mammalian bi-potential gonad is caused by the antagonistic functions of the male-promoting FGF9 and female-promoting WNT4 signalling pathways [9,29,30]. Due to the opposite sex-specific expression pattern shown by FGF9 and WNT4 in the chicken gonad, it is proposed here that the antagonistic relationship

between FGF9 and WNT4 observed in mammals is operating in the sexual development of the bi-potential chicken gonad.

13. ß-CATENIN is the effector of the WNT4-signalling pathway [31].

Finally, a comment regarding the reduction of the complete network shown in Figure S1 is pertinent here. It has been reported that ZZ AMH loss-of-function mutant gonads develop normally, expression of key testis determining genes being not altered, but the growth of the chicken embryonic urogenital tract is affected, so that the gonads of both sexes are reduced in size [32].

3. References

1. Thomas R, D'Ari R. 1990 Biological Feedback. **CRC Press**, Boca Raton, Florida, USA.

2. Chaouiya C, Remy E, Mossé B, Thieffry D. 2003 Qualitative analysis of regulatory graphs: a computational tool based on a discrete formal framework. Lecture Notes in Control and Information Science. Springer Berlin Heidelberg. **294**,119–126. (doi:10.1007/b79667)

3. Abou-Jaoudé W, Traynard P, Monteiro PT, Saez-Rodriguez J, Helikar T, Thieffry D, Chaouiya C. 2016 Logical modeling and dynamical analysis of cellular networks. Frontiers in Genetics **7**,00094. (doi: 10.3389/fgene.2016.00094)

4. Chaouiya C, Naldi A, Thieffry D. 2012 Logical Modelling of Gene Regulatory Networks with GINsim. Methods Mol. Biol. **804**, 463-479. (doi: 10.1007/978-1-61779-361-5_23)

5. Chaouiya C, Bérenguier D, Keating SM, Naldi A, van Iersel MP, Rodriguez N, Dräger A, Büchel F, Cokelaer T, Kowal B, Wicks B, Gonçalves E, Dorier J, Page M, Monteiro PT, von Kamp A, Xenarios I, de Jong H, Hucka M, Klamt S, Thieffry D, Le Novère N, Saez-Rodriguez J, Helikar T. 2013 SBML qualitative models: a model representation format and infrastructure to foster interactions between qualitative modelling formalisms and tools BMC Syst. Biol. **7**,135. (doi: 10.1186/1752-0509-7-135)

6. Thieffry D, Sánchez L. 2002 Alternative epigenetic states understood in terms of specific regulatory structures. In *Epigenesis to Epigenetics. The Genome in Context* (eds Van Speybroeck L, Van de Vijver G, De Waele D. Ann. N. Y. Acad. Sci. **981**, 135-153. (PMID:12547678)

7. Fauré A, Naldi A, Chaouiya C, Thieffy D. 2006 Dynamical analysis of a generic Boolean model for the control of the mammalian cell cycle. Bioinformatics **22**, e124-131. (doi: 10.1093/bioinformatics/btl210)

8. Bérenguier D, Chaouiya C, Monteiro PT, Naldi A, Remy E, Thieffry D, Tichit L. 2013 Dynamical modeling and analysis of large cellular regulatory networks. Chaos **23**, 025114. (doi.org/10.1063/1.4809783)

9. Sánchez L, Chaouiya C. 2016 Primary sex determination of placental mammals: a modelling study uncovers dynamical developmental constraints in the formation of Sertoli and granulosa

cells. BMC Syst. Biol. **10**, 37. (doi:10.1186/s12918-016-0282-3)

10. Naldi A, Remy E, Thieffry D, Chaouiya C. 2011 Dynamically consistent reduction of logical regulatory graphs. Theoretical Computer Science 412, 2207-2218. (doi:10.1016/j.tcs.2010.10.021.10)

11. Bagheri-Fam S, Sinclair AH, Koopman P, Harley VR. 2010 Conserved regulatory modules in the *Sox9* testis-specific enhancer predict roles for SOX, TCF/LEF, Forkhead, DMRT, and GATA proteins in vertebrate sex determination. Int. J. Biochem. Cell Biol. **42**, 472–477. (doi: 10.1016/j.biocel.2009.07.001)

12. Lambeth LS, Raymond CS, Roeszler KN, Kuroiwa A, Nakata T, Zarkower D, Smith CA. 2014 Over-expression of DMRT1 induces the male pathway in embryonic chicken gonads. Dev. Biol. **389**, 160–172. (doi: 10.1016/j.ydbio.2014.02.012)

13. Smith CA, Andrews JE, Sinclair AH. 1997 Gonadal sex differentiation in chicken embryos: Expression of estrogen receptor and aromatase genes. J. Steroid Molec. Biol. **60**, 295-302. (PMID:9219920)

14. Smith AC and Sinclair AH. 2004 Sex determination: insights from the chicken. BioEssyas **26**, 120-132. (doi:10.1002/bies.10400)

15. Smith CA, Smith MJ and Sinclair AH. 1999. Gene expression during gonadogenesis in the chicken embryo. Gene **234**, 395–402. (PMID:10395913)

16. Yoshida K, Shimada K, Saito N. 1996 Expression of P450 17αhydroxylase and P450 aromatase genes in the chicken gonad before and after sexual differentiation. Gen. Comp. Endocrinol. **102**, 233-240. (doi: 10.1006/gcen.1996.0064)

17. Oréal E, Pieau C, Mattei MG, Josso N, Picard JY, Carré-Eusèbe D, Magre S. 1998 Early expression of *amh* in chicken embryonic gonads precedes testicular *sox9* expression. Dev Dyn. **212**, 522–532. (doi: 10.1002/(SICI)1097-0177(199808)212:4<522::AID-AJA5>3.0.CO;2-J)

18. Fang L, Xin, Che Y, Xu S. 2013 Expression of Sex-Related Genes in Chicken Embryos During Male-to-Female Sex Reversal Exposure to Diethylstilbestrol. J. Integrative Agriculture **12**, 127-135. (doi: 10.1016/S2095-3119(13)60213-0).

19. Kudo T, Yamamoto H, Sato S, Sutou S. 1996 Comparison of 5’ upstream regions of chicken and quail aromatase genes. J. Reprod. Dev. **42**, 101-107. (doi: 10.1262/jrd.42.101)

20. Takada S, Wada T, Kaneda R, Choi YL, Yamashita Y, Mano H. 2006 Evidence for activation of the *Amh* gene expression by steroidogenic factor 1. Mech. Dev. **123**, 472–480. (doi: 10.1016/j.mod.2006.04.004)

21. Smith CA, Smith MJ, Sinclair AH. 1999 Expression of chicken steroidogenic factor-1 during gonadal sex differentiation. Gen. Comp. Endocrinol. **113**, 187-196. (doi: 10.1006/gcen.1998.7214)

22. Oréal E, Mazaud S, Picard JY, Magre S, Carré-Eusèbe D. 2002 Different patterns of anti-Mullerian hormone expression, as related to DMRT1, SF-1, WT1, GATA-4, Wnt-4, and Lhx9 expression, in the chick differentiating gonads. Dev. Dyn. **225**, 221–232. (doi: 10.1002/dvdy.10153)

23. Vaillant S, Magre S, Dorizzi M, Pieau C, Richard-Mercier N. 2001. Expression of *AMH*, *SF1*, and *SOX9* in gonads of genetic female chickens during sex reversal induced by an aromatase inhibitor. Dev. Dyn. **222**, 228–237. (doi: 10.1002/dvdy.1190)

24. Lambeth LS, Morris K, Ayers KL, Wise TG, O'Neil T, Wilson S, Cao Y, Sinclair AH, Cutting AD, Doran TJ, Smith CA. 2016 Overexpression of anti-Mullerian hormone disrupts gonadal sex differentiation, blocks sex hormone synthesis, and supports cell autonomous sex development in the chicken. Endocrinology **157**, 1258–1275. (doi: 10.1210/en.2015-1571)

25. Yoshioka H, Ishimaru Y, Sugiyama N, Tsunekawa N, Noce T, Kasahara M, Morohashi K. 2005 Mesonephric FGF signaling is associated with the development of sexually indifferent gonadal primordium in chick embryos. Dev. Biol. **280**,150–161. (doi: 10.1016/j.ydbio.2005.01.011)

26. Kim Y, Bingham N, Sekido R, Parker KL, Lovell-Badge R, Capel B. 2007 Fibroblast growth factor receptor 2 regulates proliferation and Sertoli differentiation during male sex determination. Proc. Natl. Acad. Sci. USA. **104**,16558–16563. (doi: 10.1073/pnas.0702581104)

27. Bagheri-Fam S, Sim H, Bernard P, Jayakody I, Taketo MM, Scherer G and Harley VR. 2008 Loss of Fgfr2 leads to partial XY sex reversal. Dev Biol. **314**,71–83. (doi: 10.1016/j.ydbio.2007.11.010)

28. Smith CA, Shoemaker CM, Roeszler KN, Queen J, Crews D, Sinclair AH. 2008 Cloning and expression of R-Spondin1 in different vertebrates suggests a conserved role in ovarian development. BMC Dev. Biol. **8**,72. (doi: 10.1186/1471-213X-8-72)

29. Kim Y, Kobayashi A, Sekido R, DiNapoli L, Brennan J, Chaboissier MC, Poulat F, Behringer RR, Lovell-Badge R, Capel B. 2006 Fgf9 and Wnt4 act as antagonistic signals to regulate mammalian sex determination. PLoS Biol. **4**,e187. (doi:10.1371/journal.pbio.0040187)

30. Kim Y, Capel B. 2006 Balancing the Bipotential Gonad Between Alternative Organ Fates: A New Perspective on an Old Problem. Dev. Dyn. **235**,2292–2300. (doi: 10.1002/dvdy.20894)

31. Tevosian SG, Manuylov NL. 2008 To ß or Not to ß: Canonical ß-Catenin Signaling Pathway and Ovarian Development. Dev. Dyn. **237**,3672–3680. (doi: 10.1002/dvdy.21784)

32. Lambeth LS, Ayers K, Cutting AD, Doran TJ, Sinclair AH, Smith CA. 2015 Anti-Mullerian hormone is required for chicken embryonic urogenital system growth but not sexual differentiation. Biol. Reprod. **93**,138. (doi: 10.1095/biolreprod.115.131664)
