# Supplementary material for: Logical modelling uncovers developmental constraints for primary sex determination of chicken gonads
Source: J R Soc Interface. 2018 May 23;15(142):20180165. doi: 10.1098/rsif.2018.0165 (PMC6000168; doi:10.1098/rsif.2018.0165)

Figure S1: Gene regulatory network controlling chicken primary sex determination. Normal green (resp. blunt red) arrows represent positive (resp. negative) interactions, and dashed arrows indicate indirect or proposed interactions. Z1 and Z2 represent each a Z chromosome, whereas W denotes a W chromosome; sex chromosome constitution (ZW or ZZ) is thus specified by an adequate combination of these input nodes.

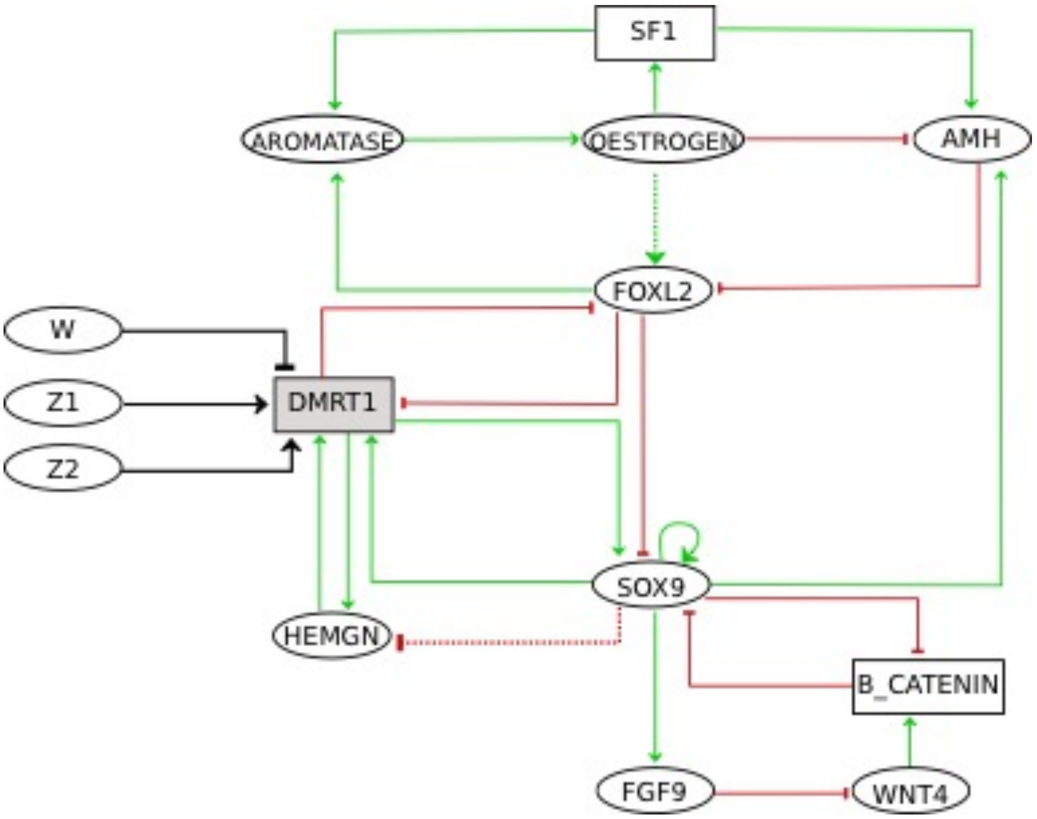

Supplement: Figure S1 [file rsif20180165supp2.pdf]
