# Supplementary material for: Logical modelling uncovers developmental constraints for primary sex determination of chicken gonads
Source: J R Soc Interface. 2018 May 23;15(142):20180165. doi: 10.1098/rsif.2018.0165 (PMC6000168; doi:10.1098/rsif.2018.0165)

Figure S2: Top: Hierarchical Transition Graphs (HTG) revealing bifurcations in the discrete dynamics and the required restrictions on the delays to ensure the reachability of the expected final stable state (see main text). Bottom: State Transition Graphs (STGs) showing the succession of events considering the proposed priority settings (see Table 1). In both graphs, the transitions are annotated with the involved events, where the signs + and -associated to the gene names indicate updates increasing and decreasing the corresponding gene levels; the vector states provide the values of the model components, in the following order: Z1, Z2, W, DMRT1, HEMGN, SOX9, FOXL2, AROMATASE, OESTROGEN, AMH.

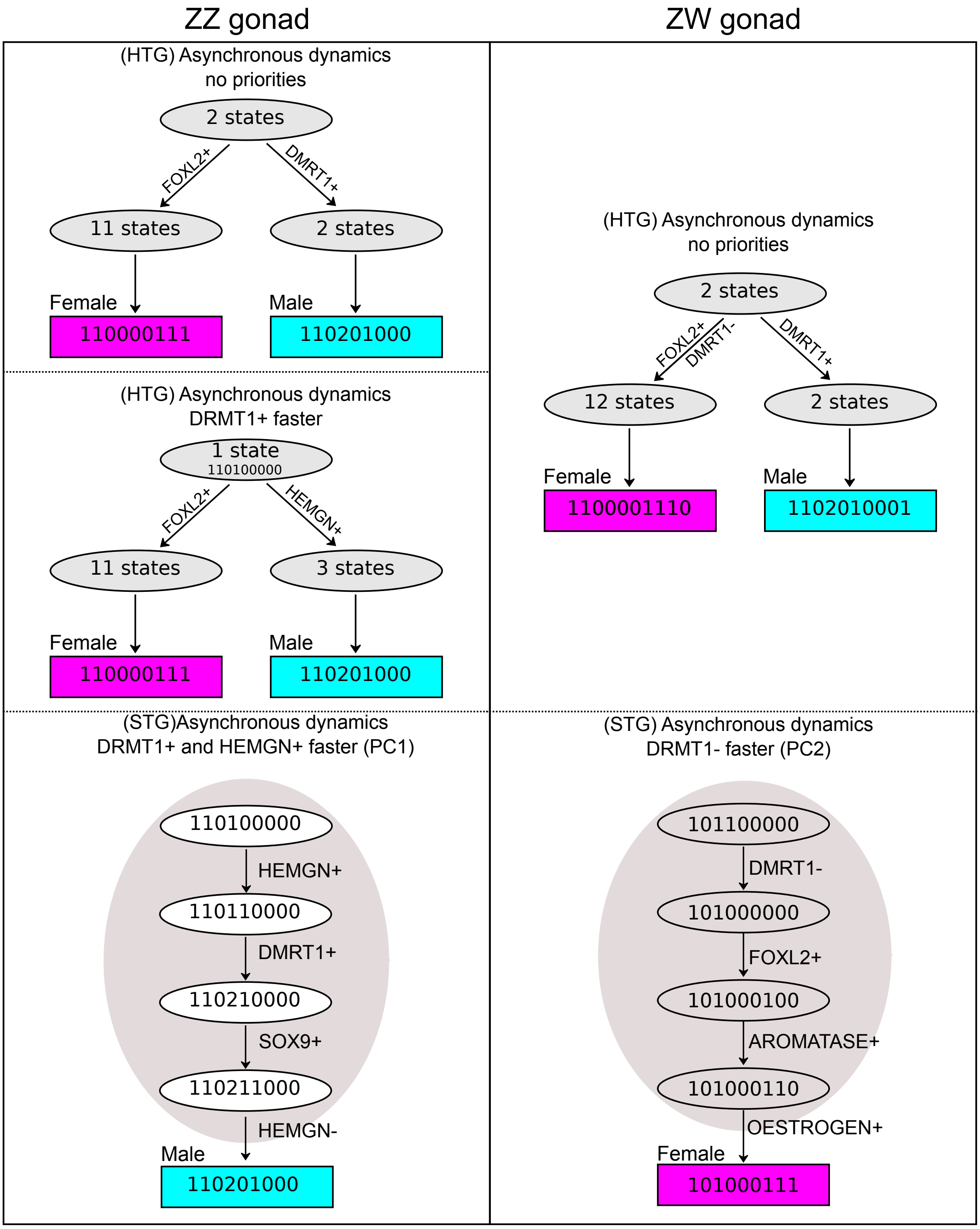

Supplement: Figure S2 [file rsif20180165supp3.pdf]
