# Supplementary material for: Logical modelling uncovers developmental constraints for primary sex determination of chicken gonads
Source: J R Soc Interface. 2018 May 23;15(142):20180165. doi: 10.1098/rsif.2018.0165 (PMC6000168; doi:10.1098/rsif.2018.0165)
Supplement: Table S1 [file rsif20180165supp4.docx]

**Table S1:** Equations defining the functional states for the genes of the network shown in Figure 2.

| **Node** | **Level** | **Logical function** |
| --- | --- | --- |
| Z1 | 1/0 | *no function (input)* |
| Z2 |  |  |
| W |  |  |
| DMRT1 | 2 | (HEMGN \| SOX9) & !FOXL2 |
|  | 1 | Z1 & Z2 & !W & !HEMGN & !FOXL2 & !SOX9 |
|  | 0 | *otherwise* |
| HEMGN | 1 | !SOX9 & DMRT1:1 |
|  | 0 | *otherwise* |
| SOX9 | 1 | (SOX9 \| DMRT1:2) & !FOXL2 |
|  | 0 | *otherwise* |
| FOXL2 | 1 | (!DMRT1:2 & !SOX9) \| (OESTROGEN & !DMRT1) |
|  | 0 | *otherwise* |
| AROMATASE | 1 | FOXL2 \| OESTROGEN |
|  | 0 | *otherwise* |
| OESTROGEN | 1 | AROMATASE |
|  | 0 | *otherwise* |
